# Epidemiology of NMOSD in Sweden from 1987 to 2013: A nationwide population-based study

**DOI:** 10.1212/WNL.0000000000007746

**Published:** 2019-07-09

**Authors:** Dagur Ingi Jonsson, Olafur Sveinsson, Ramil Hakim, Lou Brundin

**Affiliations:** From the Department of Neurology (D.I.J., O.S., L.B.), Karolinska University Hospital; and Department of Clinical Neuroscience (O.S., R.H.), Karolinska Institutet, Stockholm, Sweden.

## Abstract

**Objective:**

To report the yearly incidence rate and prevalence of neuromyelitis spectrum disorder (NMOSD) in Sweden and to investigate clinical characteristics, treatment, and outcome.

**Methods:**

We conducted a retrospective study of hospital case records of 294 individuals diagnosed with neuromyelitis optica (NMO) (G36.0 ICD-10, 341.0 ICD-9) in the Swedish National Patient Register from 1987 to end of 2013 or detected by the presence of aquaporin-4 (AQP4) immunoglobulin G (IgG) in serum during the study period. Ninety-two patients (51 NMO and 41 NMOSD) met the 2006 Wingerchuk criteria and were included in the study. Ten patients with an onset of NMO prior to 1987 and alive at the end of 2013 were included when estimating the prevalence.

**Results:**

The average yearly incidence rate per 1,000,000 individuals increased significantly from 0.30 (confidence interval [CI] 0.19–0.41) between 1987 and 2006 to 0.79 (CI 0.55–1.03) between 2007 and 2013. The prevalence was 10.4 (CI 8.5–12.6) per 1,000,000 individuals at end of 2013. The median time from onset to first relapse was 1.42 years (range 0.58–3.90). The probability of relapse was 60% and 75% after 5 and 10 years after onset. More than 80% were treated with immunosuppressive drugs. Three patients died during the study period.

**Conclusion:**

The increased incidence rate during the study period was likely due to heightened awareness and increased access to MRI and AQP4-IgG analysis. Incidence and prevalence of NMO in Sweden correspond to other countries with a predominately Caucasian population. We found that most patients were treated with immunosuppressant drugs, presumably resulting in low mortality among the detected cases.

Neuromyelitis optica (NMO)^1^ is an inflammatory demyelinating disease of the CNS.^2–12^ The primary presentation of NMO is monocular or binocular optic neuritis (ON) or longitudinally extensive transverse myelitis (LETM), area postrema, or brainstem syndrome,^3,8,10,11,13–17^ with further risk of relapses, serious disability, and not uncommonly death.^11^ NMO is more abundant among women (80%), and the typical age at onset is 35–42 years.^1,7,12,16,18–23^ A relapsing course of disease is described in 80%–90% of patients.^1,16–21,24,25^ Importantly, routine detection and measurement of NMO autoantibodies (NMO immunoglobulin G [IgG]) targeting aquaporin-4 (AQP4) receptors became available around 2007.^3,10,11,26,27^ NMO-IgG assays are highly specific (85%–99%) but only moderately sensitive (58%–76%), although methodologies have been refined.^13,26–30^ Even though NMO spectrum disorder (NMOSD) is a rare disorder, it is important to distinguish it from multiple sclerosis (MS) since treatments are different and several MS treatments may elicit relapses in NMO.^31,32^

In 2006, Wingerchuk et al.^25^ revised the criteria for the diagnosis of NMO and the introduction of NMOSD. This allowed clinicians to diagnose patients with symptoms limited to either ON or LETM by the presence of AQP4-IgG.^3,11^ The most recent 2015 diagnostic criteria for NMOSD included MRI-specific items and enabled the diagnosis of patients with the non-opticospinal presentation. Overall, the introduction of MRI and NMO-IgG antibody detection and the adjustment of the diagnostic criteria have increased the possibility of detecting NMOSD.

The incidence of NMO per 1,000,000 person-years has varied substantially, from 0.4 in Catalonia to 0.8 in the United Kingdom and central Denmark to 4 in southern Denmark.^1,9,12,22^ Although a number of epidemiologic studies on NMOSD have been conducted over recent years, many have methodologic limitations, such as few individuals in each study, limited geographical areas where representativeness is uncertain, short follow-up time, and age limitations.^9,19,22,24,33–35^ Furthermore, some studies have only attained cases through positive AQP4-IgG analysis and have not had full access to all patient records. We therefore carried out a nationwide, population-based, cohort study of the incidence and prevalence of NMO and NMOSD in all ages over a long period. In addition, we assessed clinical characteristics, treatment, and outcome.

## Methods

### Data collection

The National Swedish Patient Register (NSPR) lists all patients hospitalized (with total national coverage from 1987) or managed in hospital-based ambulatory care since 2001 in Sweden.^36^ In Sweden, the vast majority of neurologic patients are followed up and treated through the hospital system. Few patients with MS are treated only by private neurologists and we know of no such patient with NMO. Each individual's outpatient visit or hospital discharge diagnosis (ICD code) is linked to a unique personal identification number. These data were collected from individual journals of all individuals registered with a diagnosis of neuromyelitis optica (ICD-10 code G36.0 or ICD-9 code 341A) in the database of the Swedish National Board of Health and Welfare from 1987 through 2013. During the study period, analysis of NMO-IgG in Sweden was performed only at Wieslab Laboratories, Malmö, Sweden. The laboratory used the immunoblot assay until 2012; since then, a cell-based analysis has been used. From the laboratory, we received a list of all individuals, their titers, and resident hospital, who tested positive during the study period.

This study used the 2006 Wingerchuk criteria, which are the generally accepted criteria during the latter half of the study period and time of data collection. The criteria require ON and myelitis along with 2 of the following 3 supportive criteria: (1) LETM (≥3 vertebral segments in length), (2) brain MRI with normal findings or with findings not consistent with MS, and (3) AQP4-IgG seropositivity. NMOSD was defined as AQP4-IgG-seropositive status with a clinical event of LETM or ON.^11^

**Figure 1:**
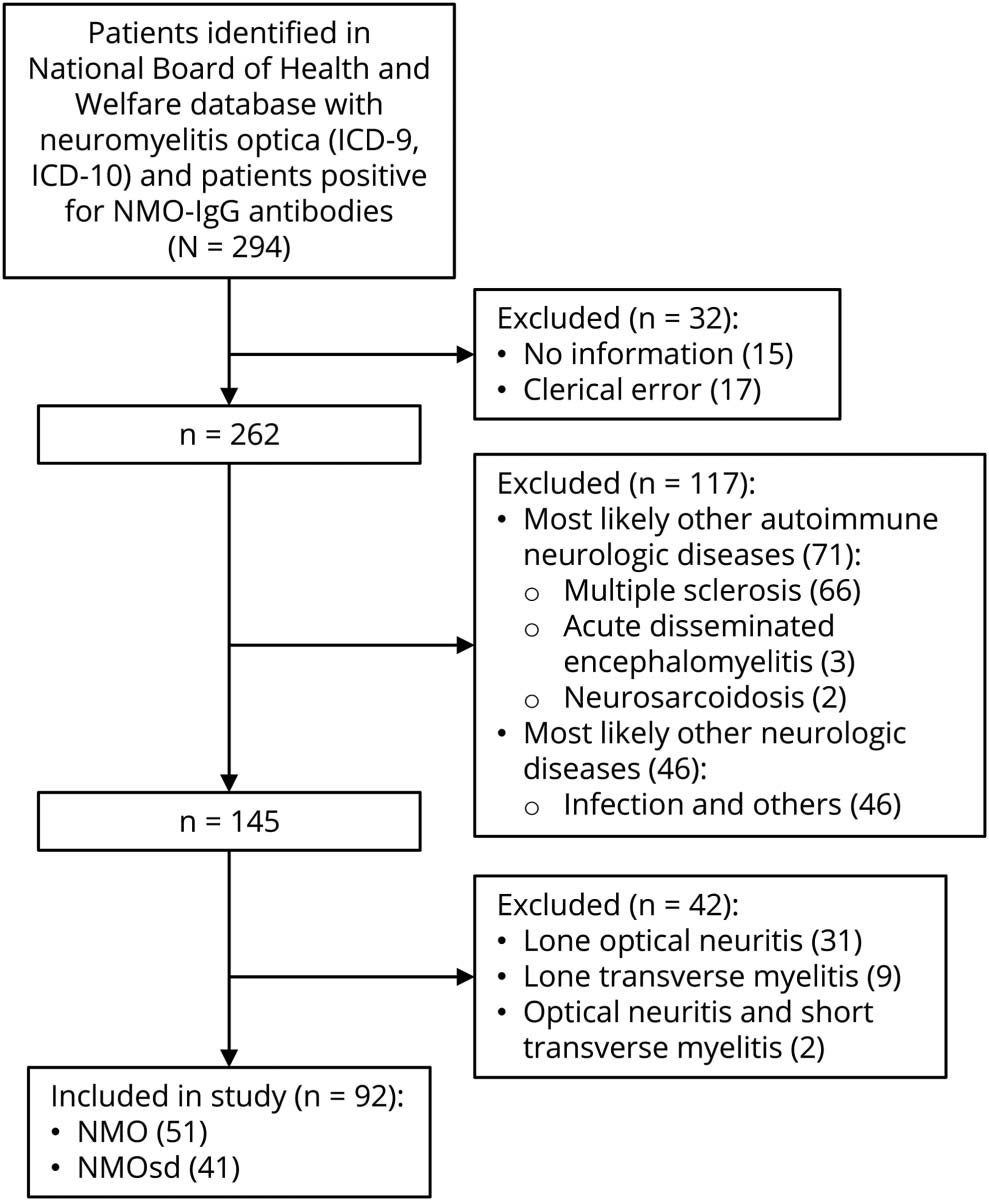
Patient selection Flowchart of exclusion and inclusion of patients for estimation of yearly incidence rate. ICD = International Classification of Diseases; IgG = immunoglobulin G; NMO = neuromyelitis optica; NMOSD = neuromyelitis optica spectrum disorder.

After receiving information from both the NSPR and the results from the only laboratory with accredited methods for AQP4 serologic determinations in Sweden, we requested patients' medical records from all clinics and hospitals. Epidemiologic data—including demographic, clinical, CSF (cell count, protein levels, and oligoclonal bands), MRI findings, treatment, and outcome—were obtained from medical records through a structured checklist by a neurology resident. No information could be retrieved for 15 patients, and 17 more records had obvious clerical errors (figure 1). Information from 262 individuals was evaluated against the 2006 Wingerchuk criteria by 2 neurologists. After scrutiny of the records, it was clear that some patients were more likely to have other neurologic disorders such as MS, acute disseminated encephalomyelitis, or infections. These were excluded, leaving 145 patients. Antibody-negative patients who had lone ON, lone transverse myelitis (TM), or ON and short TM were excluded. Following this further exclusion, 92 patients remained and were included into the study cohort for estimation of the yearly incidence rate. Fifty-one patients had classical NMO, while the remaining 41 fulfilled NMOSD criteria (figure 1). Ten patients had an onset of NMO prior to 1987. These were considered when estimating the prevalence. The ratio between NMOSD and MS in Sweden was calculated by using the last prevalence study of MS in Sweden^37^ and the number of patients with NMO alive at the end of our study period. This study was approved by the local ethical committee in Stockholm, Sweden (No. 2010/1924-31/3).

### Statistical analysis

Population data for estimation of incidence were collected from Statistics Sweden (scb.se) and United Nations (population.un.org/wpp/DataQuery/). The prevalence calculated for the date of December 31, 2013, involved the number of prevalent NMO/NMOSD cases per 1,000,000 inhabitants. A 95% confidence interval (CI) for the prevalence was estimated using methods described by Vollset.^38^ The average yearly incidence rate was defined as the number of new NMO/NMOSD cases (calculated using the date of symptom onset) for the period from January 1, 1987, to December 31, 2013, divided by the total number of people-years at risk (estimated by summing the mid-year census population of each year). This was reported per 1,000,000 people-years. Standardized incidence ratio (SIR) was estimated by adjusting for sex and age distribution and presented with a 95% CI estimated using Byar approximation. Zero estimates were taken into account when estimating SIR for time periods. Data were presented with mean and 95% CI or median with range (25th and 75th percentiles) when appropriate. The distribution of data was presented using histograms with the appropriate number of bins. Trends over time were estimated using linear regression models and reported with CIs when appropriate. The probability to first relapse was evaluated using the Kaplan-Meier method. Right censoring was implemented, and the last day of follow-up was set as December 31, 2013, as long as the patient had not relapsed. The log-rank test was utilized for comparison of relapse probability between strata. *p* Values <0.05 were considered significant. Data and statistical analysis and data presentation were prepared in R (version 3.4.4)^39^ using packages *data.table*^40^ and *ggplot2.*^41^

### Data availability

Anonymized data of this study will be available from the corresponding author on reasonable request from any qualified investigator, following the EU General Data Protection Regulation.

## Results

### Prevalence and incidence

**Figure 2:**
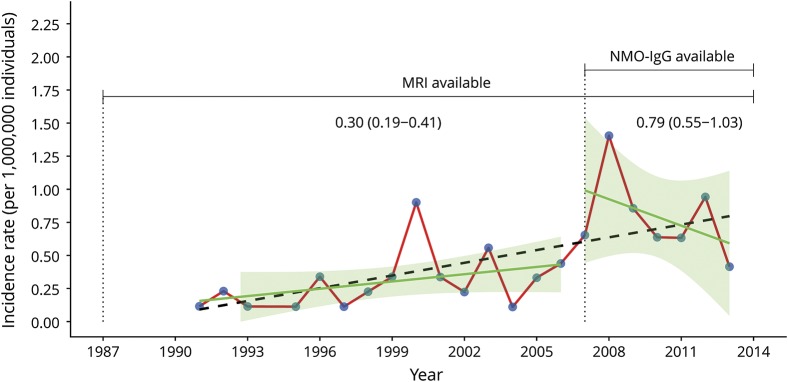
Incidence rate of neuromyelitis optica (NMO) in Sweden between 1987 and 2013 Incidence rate per 1,000,000 individuals from 1987 to 2013 in Sweden. Blue dots represent the incidence rate per year and are connected with a red line for enhanced interpretation. The availability of MRI and aquaporin-4–immunoglobulin G (IgG) autoantibody analysis are reported in the figure. The black dotted line represents a linear regression model (incidence rate per 1,000,000 individuals = 0.03205 * year − 63.7) fitted to all data points. Green lines and green shaded area represent linear regression models with 95% confidence intervals fitted within periods 1987 to 2006 and 2007 to 2013, respectively. Values in plot are means with 95% confidence intervals for the time intervals 1987 to 2006 and 2007 to 2013.

The prevalence of NMO/NMOSD in Sweden as of 2013 was 9.3 (CI 7.6–11.5) individuals per 1,000,000 individuals. After standardization to the world population by age, the prevalence was 1.03 (CI 0.66–1.55) for males and 0.99 (CI 0.77–1.26) for females. The ratio between NMO/NMOSD and MS in Sweden based on prevalence was calculated to be 100 vs 17,485 (as of 2008)^37^ or a ratio of 1 NMO patient for every 175 MS patients (0.6%). The yearly crude incidence rate showed an increasing trend over time from 1987 to 2013 (figure 2). Age-standardized estimates of yearly incidence rate for each sex are available in table e-1 (doi.org/10.5061/dryad.vk62410). Analysis of AQP4-IgG became available in 2007. The yearly crude incidence rate for NMO/NMOSD increased from 1987 to 2006 (0.23, range 0.12–0.34) to 2007–2013 (0.65, range 0.63–0.90) (table e-2, doi.org/10.5061/dryad.vk62410). Although the crude incidence rate increased for both NMO and NMOSD, the crude rate for NMOSD increased slightly more than for NMO. No significant increase in incidence could be detected among males between 1987–2006 and 2007–2013 when comparing SIR retrieved by adjusting for sex and age (table 1). The incidence did, however, increase significantly for females from 1987 to 2006 (0.7, CI 0.4–0.9) to 2007–2013 (1.50, CI 1.1–2.0) as detected comparing SIR adjusted for age (table 1).

**Table 1:**
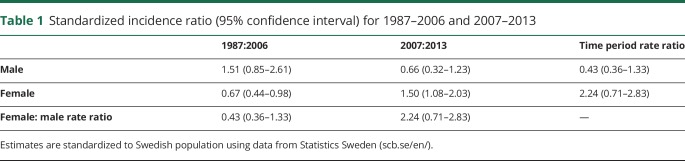
Standardized incidence ratio (95% confidence interval) for 1987–2006 and 2007–2013

### Demography, clinical characteristics, treatment, and outcome

**Figure 3:**
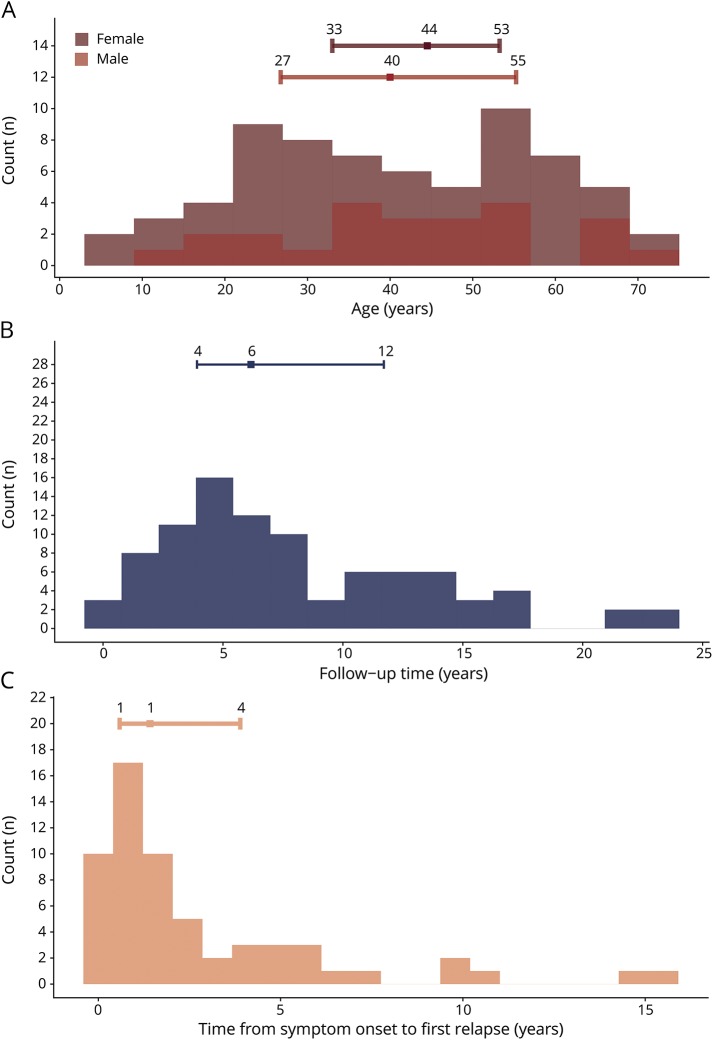
Patient characteristics Distributions of continuous demographic variables. Figure reports histograms for age (A), follow-up time (B), and time from symptom onset to relapse (C) in years. Number of bins used varies between histograms. Error bars represent median surrounded by 25th and 75th percentiles. Exact values are given in relation to the relevant position along the error bars.

The majority of patients in the cohort were female (73.9%) (table 2). The patients with NMOSD were on average >10 years older (47.7, CI 42.7–52.8) than patients with NMO (36.4, CI 32.0–40.9). The age distribution was even around a mean of 41.5 for both males and females (figure 3A). Ten individuals (9.7%) were non-Caucasian. Autoimmune comorbidities were detected in 25.0%. Among the NMOSD group, 36.6% had ON and 63.4% LETM, respectively. Bulbar symptoms were found in 15.2% of all patients. No difference could be detected between patients with NMO and NMOSD in terms of findings of elevated leukocytes in CSF or the presence of oligoclonal bands. The most common drug given was corticosteroid treatment (87.0% received steroids at least once). Azathioprine and rituximab were commonly used as disease-modifying drugs (44.6% and 38.0%, respectively). Four (4.3%) patients underwent autologous hematopoietic stem cell transplantation, mainly due to previous treatment failure. The median follow-up time was 6.2 years (range 3.9–11.7) (figure 3B). Two patients with a disease onset after 1987 and one with an onset prior to 1987 died during the study period (3.3%).

**Table 2:**
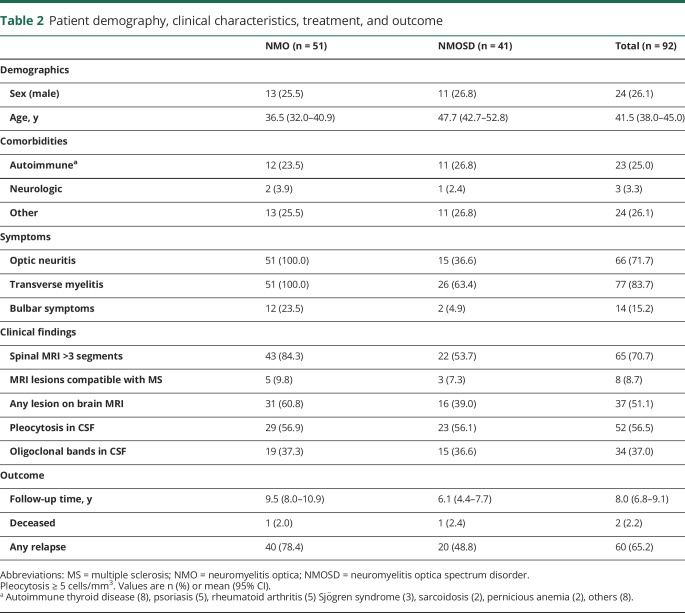
Patient demography, clinical characteristics, treatment, and outcome

### Relapses

**Figure 4:**
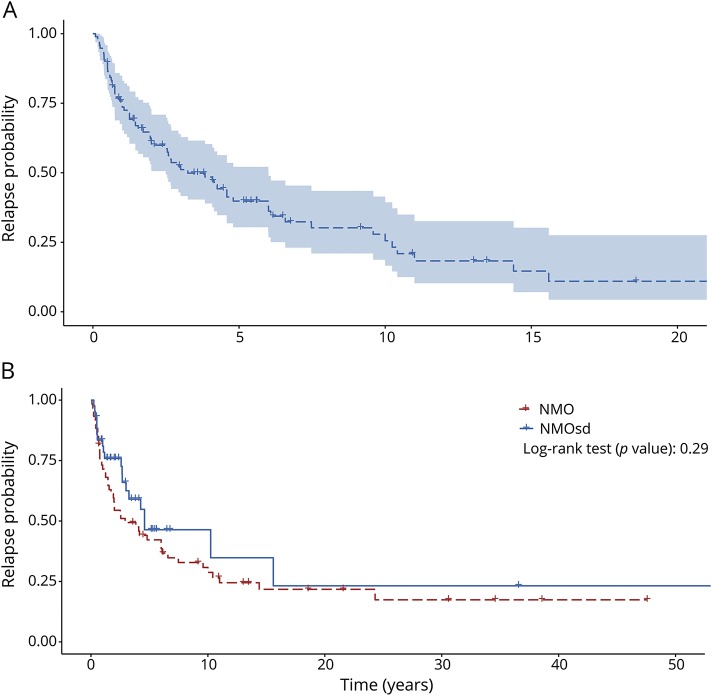
Relapse probability Relapse probability for all patients (A) and divided by neuromyelitis optica (NMO)/neuromyelitis optica spectrum disorder (NMOSD) (B). (A) Kaplan-Meier curve surrounded by a 95% confidence interval. Zero time is onset of NMO/NMOSD. Time from onset to relapse is reported in years. (B) *p* Value of a log-rank test between the relapse curves.

More than half (65.2%) of the patients relapsed at least once during the follow-up period. The time from onset to a first relapse was left skewed with a median time of 1.42 years (range 0.6–3.9) (figure 3C). A majority (78.4%) of the patients with NMO relapsed, while half (48.8%) of the patients with NMOSD relapsed; this difference was not significant (*p* > 0.05) (figure 4B). Using the Kaplan-Meier estimator and right censoring, half of the patients relapsed within the first 5 years of onset (figure 4A). On the other hand, the long-term probability for relapse was about 80% during the 26-year follow-up period. The symptom at onset (ON, TM, or both) did not affect the probability of a new relapse (*p* > 0.05).

## Discussion

In this study, we sought to determine the average yearly incidence rate and the current prevalence of NMO and NMOSD in Sweden. The average yearly incidence rate of NMO/NMOSD was 0.79 (CI 0.55–1.03), and the prevalence was 10.4 patients per million individuals as of the end of 2013. The incidence rate increased steadily from 1987 to 2013, most likely because of increased vigilance but also due to greater usage of diagnostic methods such as MRI (∼1990 onwards) and analysis of AQP4-IgG serostatus (2007 onwards).

Although the study is nationwide and population-based, the estimated incidence and prevalence are to be considered minimum estimates. It is known that estimates of incidence and prevalence of rare diseases using registry data are likely to be lower than their true equivalents.^42^ Furthermore, we know from clinical experience that patients with NMOSD who do not present with extensive myelitis are at risk of being incorrectly diagnosed with MS. A patient with extensive myelitis is more likely to be tested for AQP4 serostatus and correctly identified as NMOSD than an individual with shorter myelitis or an isolated ON. On the other hand, the high accessibility of publicly funded health care in Sweden in combination with the debilitating symptoms of NMOSD make it unlikely that patients remain unnoticed or receive treatment at private institutions.

**Figure 5:**
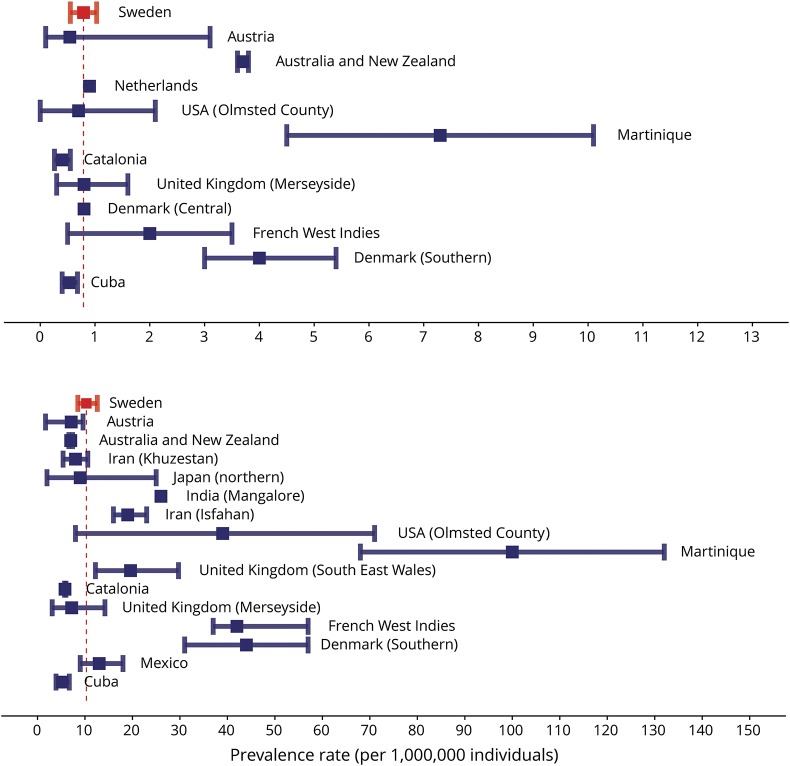
Comparison of incidence and prevalence in Sweden with other regions Incidence (A) and prevalence (B) of neuromyelitis optica/neuromyelitis optica spectrum disorder in other regions from available publications. Horizontal error bars represent the mean surrounded by a 95% confidence interval.

The average incidence rate in Sweden did not differ from the incidence rate in central Denmark, Merseyside (UK), or Cuba, was slightly higher than the rate in Catalonia, but was much lower than the rate in southern Denmark (figure 5A). The prevalence in Sweden was estimated at 10.37 patients with NMO per million individuals, which reflects the prevalence in South East Wales (UK), Merseyside (UK), and Mexico, but is higher than the prevalence in Catalonia and Cuba and lower than the prevalence in southern Denmark (figure 5B).^1,7,9,12,19,22,24,33–35,43–49^ Thus, the incidence and prevalence of NMO/NMOSD in Sweden is comparable to data reported from countries with a predominately Caucasian population. Approximately 90% of the patients included in this study were of Caucasian origin, which is similar to the Swedish population. Our data, however, do not correspond to Southern Denmark^1^ or the United States (Olmsted County)^43^ (figure 5). In these 2 studies, all patients who presented with demyelinating disease^42^ or an episode of ON or TM and an initial brain MRI not diagnostic for MS^1^ were screened for AQP4-IgG and were probably diagnosed earlier and more accurately. Because the demographics are similar in Sweden and Denmark, the absence of such a strategy in Sweden suggests that several cases of NMOSD were not detected. This underpins the importance of screening patients with a demyelinating event for AQP4-IgG serostatus.

In this study, autoimmune comorbidities were detected in 25.0%, which is comparable to another recent population-based study from Catalonia (27%).^12^ We also had very similar female to male ratio (3/1) and average age at onset (41.5 years) as the Catalan study. Other studies have described a higher female to male ratio (up to 9/1 in Martinique)^43^ and younger average age at onset,^9,23,43^ down to 31 years in Iran.^7^

We could not detect any clinical difference between patients with NMO and NMOSD regarding imaging findings, level of leukocytes in CSF, or presence of oligoclonal bands. This supports the increased evidence that NMO and NMOSD follow a similar clinical course and should be regarded as the same disease. To our surprise, the patients with NMO were, on average, 10 years younger than the patients with NMOSD. We have no explanation for this finding. Another important finding is that the type of symptoms at onset did not affect the probability of a new relapse. To our knowledge, this has not been reported before. As expected, the majority of patients with NMO/NMOSD did relapse, and the probability of relapse measured using the Kaplan-Meier estimator was not different between NMO and NMOSD. In a recent study of pretreatment annualized relapse rate, the relapse rate was 2.26–2.29.^50^ In our cohort, the risk for a first relapse was highest during the first 2 years (median 1.42 years). Also, the risk of relapses never disappeared during the study period. Considering the continual risk and severity of NMO relapses, this is in agreement with the prevailing opinion that immunosuppressive drugs should be administered and not discontinued.

To allow for early treatment of NMO/NMOSD and to reduce the risk of disability and death, correct and early diagnosis of NMO/NMOSD and distinction from MS is of utmost importance. In earlier reports, more than 50% of patients with NMO/NMOSD were blind in one or both eyes or required ambulatory help within 5 years of disease onset.^21^ In 1999, before the spectrum of NMO was understood, the calculated 5-year survival rate for NMO was 68%, with all deaths caused by neurogenic respiratory failure.^10^ The low mortality (3.3%) observed in our sample might be explained by a high use of immune-modulating drugs. Furthermore, since this was a nationwide study, it is possible that patients with a mild disease course not needing tertiary hospital care were included, thus contributing to the low mortality.

The main limitations of our study are the retrospective design and the dependency on clinicians' diagnosis or suspicion of NMO/NMOSD, which most likely contributes to underestimating the incidence and prevalence of NMO/NMOSD. Another limitation is the fact that we did not have access to records from outpatient care that was not hospital-based. Some milder cases of NMOSD may have been missed. On the other hand, the health system in Sweden is built up in such a way that fewer than 5% of neurologic outpatients are seen by private practicing neurologists. The rule is that all MS/NMO patients should be seen through the hospital system. Furthermore, since the experience of demyelinating diseases varied between clinics and systematic clinical evaluation, we were unable to retrieve and evaluate the Expanded Disability Status Scale score for the majority of patients.

Even though the incidence and prevalence of NMO/NMOSD in Sweden are in line with most reports from countries with a predominately Caucasian population, we still suspect that NMOSD in Sweden is underdiagnosed, which could be compensated by increased vigilance and routine AQP4-IgG antibody analysis. More than 80% of patients were treated with disease-inhibiting immunosuppressive drugs (mostly azathioprine and rituximab), which possibly affected the low mortality rate among detected cases. We found a high risk of relapse within a fairly short time; considering the severity of relapses, this should be kept in mind when counselling a patient with NMOSD. All patients should receive continuous disease-inhibiting immunosuppressive treatment. An initiative for systematic prospective national follow-up has recently been initiated through the Swedish registry for neurologic diseases. It is our hope that it will reach full national coverage within a limited time frame.

## Glossary

AQP4: aquaporin-4
CI: confidence interval
ICD: International Classification of Diseases
IgG: immunoglobulin G
LETM: longitudinally extensive transverse myelitis
MS: multiple sclerosis
NMO: neuromyelitis optica
NMOSD: neuromyelitis optica spectrum disorder
NSPR: National Swedish Patient Register
ON: optic neuritis
SIR: standardized incidence ratio
TM: transverse myelitis

## References

[1] Asgari N, Lillevang ST, Skejoe HP, Falah M, Stenager E, Kyvik KO. A population-based study of neuromyelitis optica in Caucasians. Neurology 2011;76:1589–1595. 21536639 10.1212/WNL.0b013e3182190f74 PMC3269768

[2] Lucchinetti CF, Mandler RN, McGavern D, et al. A role for humoral mechanisms in the pathogenesis of Devic's neuromyelitis optica. Brain 2002;125:1450–1461. 12076996 10.1093/brain/awf151 PMC5444467

[3] Wingerchuk DM, Lennon VA, Lucchinetti CF, Pittock SJ, Weinshenker BG. The spectrum of neuromyelitis optica. Lancet Neurol 2007;6:805–815. 17706564 10.1016/S1474-4422(07)70216-8

[4] Jacob A, Matiello M, Wingerchuk DM, Lucchinetti CF, Pittock SJ, Weinshenker BG. Neuromyelitis optica: changing concepts. J Neuroimmunol 2007;187:126–138. 17512987 10.1016/j.jneuroim.2007.04.009

[5] Mealy MA, Wingerchuk DM, Greenberg BM, Levy M. Epidemiology of neuromyelitis optica in the United States: a multicenter analysis. Arch Neurol 2012;69:1176–1180. 22733096 10.1001/archneurol.2012.314

[6] Jarius S, Ruprecht K, Wildemann B, et al. Contrasting disease patterns in seropositive and seronegative neuromyelitis optica: a multicentre study of 175 patients. J Neuroinflammation 2012;9:14. 22260418 10.1186/1742-2094-9-14 PMC3283476

[7] Kashipazha D, Mohammadianinejad SE, Majdinasab N, Azizi M, Jafari M. A descriptive study of prevalence, clinical features and other findings of neuromyelitis optica and neuromyelitis optica spectrum disorder in Khuzestan Province, Iran. Iranian J Neurol 2015;14:204–210. PMC4754599 26885339

[8] Jasiak-Zatonska M, Kalinowska-Lyszczarz A, Michalak S, Kozubski W. The immunology of neuromyelitis optica: current knowledge, clinical implications, controversies and future perspectives. Int J Mol Sci 2016;17:273. 26950113 10.3390/ijms17030273 PMC4813137

[9] Jacob A, Panicker J, Lythgoe D, et al. The epidemiology of neuromyelitis optica amongst adults in the Merseyside county of United Kingdom. J Neurol 2013;260:2134–2137. 23689970 10.1007/s00415-013-6926-y

[10] Wingerchuk DM, Hogancamp WF, O'Brien PC, Weinshenker BG. The clinical course of neuromyelitis optica (Devic's syndrome). Neurology 1999;53:1107–1114. 10496275 10.1212/wnl.53.5.1107

[11] Wingerchuk DM, Lennon VA, Pittock SJ, Lucchinetti CF, Weinshenker BG. Revised diagnostic criteria for neuromyelitis optica. Neurology 2006;66:1485–1489. 16717206 10.1212/01.wnl.0000216139.44259.74

[12] Sepúlveda M, Aldea M, Escudero D, et al. Epidemiology of NMOSD in Catalonia: influence of the new 2015 criteria in incidence and prevalence estimates. Mult Scler Epub 2017 Oct 1. 10.1177/1352458517735191 28984163

[13] Waters PJ, Pittock SJ, Bennett JL, Jarius S, Weinshenker BG, Wingerchuk DM. Evaluation of aquaporin-4 antibody assays. Clin Exp Neuroimmunol 2014;5:290–303. 27840658 10.1111/cen3.12107 PMC5102503

[14] Weinshenker BG, Wingerchuk DM, Vukusic S, et al. Neuromyelitis optica IgG predicts relapse after longitudinally extensive transverse myelitis. Ann Neurol 2006;59:566–569. 16453327 10.1002/ana.20770

[15] Sellner J, Boggild M, Clanet M, et al. EFNS guidelines on diagnosis and management of neuromyelitis optica. Eur J Neurol 2010;17:1019–1032. 20528913 10.1111/j.1468-1331.2010.03066.x

[16] de Seze J, Stojkovic T, Ferriby D, et al. Devic's neuromyelitis optica: clinical, laboratory, MRI and outcome profile. J Neurol Sci 2002;197:57–61. 11997067 10.1016/s0022-510x(02)00043-6

[17] O'Riordan JI, Gallagher HL, Thompson AJ, et al. Clinical, CSF, and MRI findings in Devic's neuromyelitis optica. J Neurol Neurosurg Psychiatry 1996;60:382–387. 8774400 10.1136/jnnp.60.4.382 PMC1073888

[18] Bichuetti DB, Oliveira EM, Souza NA, Rivero RL, Gabbai AA. Neuromyelitis optica in Brazil: a study on clinical and prognostic factors. Mult Scler 2009;15:613–619. 19299436 10.1177/1352458508101935

[19] Cabre P, Heinzlef O, Merle H, et al. MS and neuromyelitis optica in Martinique (French West Indies). Neurology 2001;56:507–514. 11222796 10.1212/wnl.56.4.507

[20] Papais-Alvarenga RM, Miranda-Santos CM, Puccioni-Sohler M, et al. Optic neuromyelitis syndrome in Brazilian patients. J Neurol Neurosurg Psychiatry 2002;73:429–435. 12235313 10.1136/jnnp.73.4.429 PMC1738088

[21] Wingerchuk DM, Weinshenker BG. Neuromyelitis optica: clinical predictors of a relapsing course and survival. Neurology 2003;60:848–853. 12629245 10.1212/01.wnl.0000049912.02954.2c

[22] Dale GH, Svendsen KB, Gjelstrup MC, et al. Incidence of neuromyelitis optica spectrum disorder in the central Denmark region. Acta Neurol Scand 2018;137:582–588. 29359475 10.1111/ane.12903

[23] Collongues N, Marignier R, Zephir H, et al. Neuromyelitis optica in France: a multicenter study of 125 patients. Neurology 2010;74:736–742. 20194912 10.1212/WNL.0b013e3181d31e35

[24] Rivera JF, Kurtzke JF, Booth VJ, Corona VT. Characteristics of Devic's disease (neuromyelitis optica) in Mexico. J Neurol 2008;255:710–715. 18283393 10.1007/s00415-008-0781-2

[25] Wingerchuk DM, Banwell B, Bennett JL, et al. International consensus diagnostic criteria for neuromyelitis optica spectrum disorders. Neurology 2015;85:177–189. 26092914 10.1212/WNL.0000000000001729 PMC4515040

[26] Lennon VA, Wingerchuk DM, Kryzer TJ, et al. A serum autoantibody marker of neuromyelitis optica: distinction from multiple sclerosis. Lancet 2004;364:2106–2112. 15589308 10.1016/S0140-6736(04)17551-X

[27] Lennon VA, Kryzer TJ, Pittock SJ, Verkman AS, Hinson SR. IgG marker of optic-spinal multiple sclerosis binds to the aquaporin-4 water channel. J Exp Med 2005;202:473–477. 16087714 10.1084/jem.20050304 PMC2212860

[28] Fazio R, Malosio ML, Lampasona V, et al. Anti-aquaporin 4 antibodies detection by different techniques in neuromyelitis optica patients. Mult Scler 2009;15:1153–1163. 19667009 10.1177/1352458509106851

[29] Waters P, Vincent A. Detection of anti-aquaporin-4 antibodies in neuromyelitis optica: current status of the assays. Int MS J 2008;15:99–105. 18808744

[30] Waters P, Reindl M, Saiz A, et al. Multicentre comparison of a diagnostic assay: aquaporin-4 antibodies in neuromyelitis optica. J Neurol Neurosurg Psychiatry 2016;87:1005–1015. 27113605 10.1136/jnnp-2015-312601 PMC5013123

[31] Min JH, Kim BJ, Lee KH. Development of extensive brain lesions following fingolimod (FTY720) treatment in a patient with neuromyelitis optica spectrum disorder. Mult Scler 2012;18:113–115. 22146605 10.1177/1352458511431973

[32] Gelfand JM. Multiple sclerosis: diagnosis, differential diagnosis, and clinical presentation. Handb Clin Neurol 2014;122:269–290. 24507522 10.1016/B978-0-444-52001-2.00011-X

[33] Kuroiwa Y, Igata A, Itahara K, Koshijima S, Tsubaki T. Nationwide survey of multiple sclerosis in Japan: clinical analysis of 1,084 cases. Neurology 1975;25:845–851. 1172207 10.1212/wnl.25.9.845

[34] Cabrera-Gómez JA, Kurtzke JF, González-Quevedo A, Lara-Rodríguez R. An epidemiological study of neuromyelitis optica in Cuba. J Neurol 2009;256:35–44. 19224310 10.1007/s00415-009-0009-0

[35] Cossburn M, Tackley G, Baker K, et al. The prevalence of neuromyelitis optica in South East Wales. Eur J Neurol 2012;19:655–659. 21967235 10.1111/j.1468-1331.2011.03529.x

[36] Ludvigsson JF, Andersson E, Ekbom A, et al. External review and validation of the Swedish national inpatient register. BMC Public Health 2011;11:450. 21658213 10.1186/1471-2458-11-450 PMC3142234

[37] Ahlgren C, Odén A, Lycke J. High nationwide prevalence of multiple sclerosis in Sweden. Mult Scler 2011;17:901–908. 21459810 10.1177/1352458511403794

[38] Vollset SE. Confidence intervals for a binomial proportion. Stat Med 1993;12:809–824. 8327801 10.1002/sim.4780120902

[39] Team RC. R: A Language and Environment for Statistical Computing. Vienna: R Foundation for Statistical Computing; 2016.

[40] Dowle M, Srinivasan A. data.table: Extension of data.frame. R package version 1.12.2; 2019. Available at: https://CRAN.R-project.org/package=data.table.

[41] Wickham H. ggplot2: Elegant Graphics for Data Analysis. New York: Springer-Verlag; 2009.

[42] Richesson R, Vehik K. Patient registries: utility, validity and inference. Adv Exp Med Biol 2010;686:87–104. 20824441 10.1007/978-90-481-9485-8_6

[43] Flanagan EP, Cabre P, Weinshenker BG, et al. Epidemiology of aquaporin-4 autoimmunity and neuromyelitis optica spectrum. Ann Neurol 2016, 79:775–783. 26891082 10.1002/ana.24617 PMC4988933

[44] Etemadifar M, Dashti M, Vosoughi R, Abtahi SH, Ramagopalan SV, Nasr Z. An epidemiological study of neuromyelitis optica in Isfahan. Mult Scler 2014;20:1920–1922. 24948686 10.1177/1352458514537699

[45] van Pelt ED, Wong YY, Ketelslegers IA, Hamann D, Hintzen RQ. Neuromyelitis optica spectrum disorders: comparison of clinical and magnetic resonance imaging characteristics of AQP4-IgG versus MOG-IgG seropositive cases in The Netherlands. Eur J Neurol 2016;23:580–587. 26593750 10.1111/ene.12898

[46] Pandit L, Kundapur R. Prevalence and patterns of demyelinating central nervous system disorders in urban Mangalore, South India. Mult Scler 2014;20:1651–1653. 24493471 10.1177/1352458514521503

[47] Houzen H, Niino M, Hirotani M, et al. Increased prevalence, incidence, and female predominance of multiple sclerosis in northern Japan. J Neurol Sci 2012;323:117–122. 22995683 10.1016/j.jns.2012.08.032

[48] Bukhari W, Prain KM, Waters P, et al. Incidence and prevalence of NMOSD in Australia and New Zealand. J Neurol Neurosurg Psychiatry 2017;88:632–638. 28550069 10.1136/jnnp-2016-314839

[49] Aboul-Enein F, Seifert-Held T, Mader S, et al. Neuromyelitis optica in Austria in 2011: to bridge the gap between neuroepidemiological research and practice in a study population of 8.4 million people. PLoS One 2013;8:e79649. 24223985 10.1371/journal.pone.0079649 PMC3818238

[50] Mealy MA, Wingerchuk DM, Palace J, Greenberg BM, Levy M. Comparison of relapse and treatment failure rates among patients with neuromyelitis optica: multicenter study of treatment efficacy. JAMA Neurol 2014;71:324–330. 24445513 10.1001/jamaneurol.2013.5699

